# Tailoring the Dopant Distribution in ZnO:Mn Nanocrystals

**DOI:** 10.1038/s41598-019-43388-z

**Published:** 2019-05-03

**Authors:** Daniela Ghica, Ioana D. Vlaicu, Mariana Stefan, Valentin A. Maraloiu, Alexandra C. Joita, Corneliu Ghica

**Affiliations:** National Institute of Materials Physics, Atomistilor Str. 405A, Magurele, 077125 Romania

**Keywords:** Materials science, Materials science, Nanoparticles, Nanoparticles

## Abstract

The synthesis of semiconductor nanocrystals with controlled doping is highly challenging, as often a significant part of the doping ions are found segregated at nanocrystals surface, even forming secondary phases, rather than incorporated in the core. We have investigated the dopant distribution dynamics under slight changes in the preparation procedure of nanocrystalline ZnO doped with manganese in low concentration by electron paramagnetic resonance spectroscopy, paying attention to the formation of transient secondary phases and their transformation into doped ZnO. The acidification of the starting solution in the co-precipitation synthesis from nitrate precursors lead to the decrease of the Mn^2+^ ions concentration in the core of the ZnO nanocrystals and their accumulation in minority phases, until ~79% of the Mn^2+^ ions were localized in a thin disordered shell of zinc hydroxynitrate (ZHN). A lower synthesis temperature resulted in polycrystalline Mn-doped ZHN. Under isochronal annealing up to 250 °C the bulk ZHN and the minority phases from the ZnO samples decomposed into ZnO. The Mn^2+^ ions distribution in the annealed nanocrystals was significantly altered, varying from a uniform volume distribution to a preferential localization in the outer layers of the nanocrystals. Our results provide a synthesis strategy for tailoring the dopant distribution in ZnO nanocrystals for applications ranging from surface based to ones involving core properties.

## Introduction

One of the most studied semiconductors of the past decade, nanocrystalline zinc oxide (nano-ZnO) doped with transition metal ions continues to hold a great interest for the researchers in materials science, as changing the nature and concentration of the dopant ion is a well-tested method to tailor the material properties for a wide range of applications in spintronics, nano- and/or optoelectronics^1–4^.

However, doping colloidal semiconductor nanocrystals is still an experimental challenge^5–11^, the main issues being the control of the concentration level and distribution uniformity of the incorporated dopant. Whether the doping ions are incorporated or remain outside the nanocrystals core, they influence the crystal growth, initiating and controlling morphological and even structural changes^11–15^. As previously shown, sometimes only a small fraction of dopant ions is actually present inside the nanocrystals, the largest part being localized in the intergrain region, often in secondary phases^8,10,16–18^. In many cases, at lower dopant concentration levels (below 1 at%), these secondary phases are found as a thin amorphous shell containing isolated dopant ions^15,19^. For larger dopant concentrations impurity-rich secondary phases are formed which affect the magnetic, electrical and optical properties of the nanostructured semiconductors^11,20–26^. The amount and composition of the material in the intergrain region is strongly dependent on the synthesis conditions, which opens interesting possibilities to tailor the semiconductor nanostructures for applications requiring special surface properties (catalysis, gas sensing, etc.) or different optical, electrical and/or magnetic characteristics^23,27–29^. In order to achieve this level of control we would also need to strengthen our understanding of the doping mechanism at nanoscale, especially of the influence of the synthesis parameters and precursors on the dopant localization and doping efficiency.

The ability to determine the spatial distribution of low concentrations (below 1 at%) of doping ions in nanostructured materials, as well as the accuracy of these determinations, are yet another experimental challenge. Advanced techniques of chemical mapping at nanometric scale or even atomic spatial resolution by aberration-corrected STEM (scanning transmission electron microscopy) and EELS (electron energy loss spectroscopy) mapping^30–33^ and/or electron tomography^34–36^ provide spectacular results in imaging the local distribution of chemical elements. More recently, atom probe tomography (APT) boosted the knowledge in the field of the chemical elements distribution and segregation phenomena at atomic scale^33,37,38^. Still, all these state-of-the-art techniques have a strong local character, being less statistically relevant and not always suitable for dopant concentrations lower than a few percent.

Alternatively, electron paramagnetic resonance (EPR) spectroscopy offers a sensitive and statistically reliable method to determine the distribution/localization of *paramagnetic* dopants present in low concentrations in nanostructured semiconductors^10,15–17,19,39–46^.

The present work is focused on the assessment and control of the dopant distribution in nanocrystalline ZnO doped with a low concentration (0.1 at%) of Mn^2+^ ions (nano-ZnO:Mn) prepared by co-precipitation from nitrate precursors followed by mild thermal treatments (annealing for 10 minutes at temperatures up to 250 °C). We aim to demonstrate a simple and cost-effective method to tailor the dopant distribution of the ZnO nanocrystals for specific applications. An important part of this approach refers to the control of the amount and chemical composition of the secondary intermediate phases, expected to contain a non-negligible concentration of dopant Mn^2+^ ions, by minor adjustments of the synthesis route. These secondary phases were analysed by EPR and Fourier transform infrared (FTIR) spectroscopies combined with microstructural investigations. The dynamics of the dopant distribution under mild isochronal thermal treatments was monitored by EPR. By manipulating the secondary phases, we were able to obtain ZnO nanocrystals with tailored dopant distributions, from uniform to dopant-rich surfaces.

## Results

### X-ray diffraction (XRD) results

In order to assess the influence of the synthesis parameters on the dopant distribution and the formation of intermediate secondary phases, we have prepared several samples following a similar synthesis route, but varying one synthesis parameter from sample to sample.

**Figure 1:**
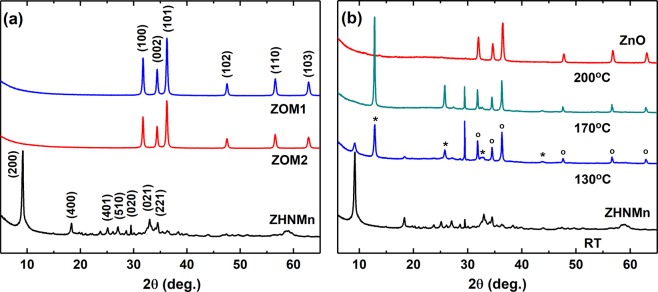
(**a**) XRD patterns of the ZOM1, ZOM2 and ZHNMn samples indexed according to the ZnO and Zn_5_(OH)_8_(NO_3_)_2_·2H_2_O structures, respectively. Only the main peaks are indicated for the ZHNMn sample. (**b**) XRD patterns of the ZHNMn sample, as-prepared (RT) and after isochronal annealing at the indicated temperatures. The ZnO phase is marked with open circles, while the Zn_3_(OH)_4_(NO_3_)_2_ phase is marked with stars.

First, we have prepared a series of five nano-ZnO:Mn samples using different amounts of nitric acid to acidify the starting solution. These samples exhibit similar XRD patterns, indexed as single phase ZnO - wurtzite (hexagonal structure, space group P6_3_mc, JCPDS 89–1397) with lattice parameters *a* = 3.254(3) Å, *c* = 5.214(5) Å. The calculated average crystallite size varies with the amount of nitric acid added during the synthesis from 38 ± 3 nm in the sample called ZOM1 to 25 ± 2 nm in the sample called ZOM2, corresponding to the smallest (50 mL) and largest (250 mL) amount of nitric acid, respectively. The XRD patterns of these two samples, selected for further spectroscopic and microstructural investigations, are presented in Fig. 1(a).

The second synthesis parameter that we changed was the synthesis temperature. Another sample was prepared by following the same synthesis steps as for ZOM2, but lowering the synthesis temperature from 80 °C to 35 °C. The XRD pattern of this sample (Fig. 1a, bottom) was indexed as pure, well crystallized Zn_5_(OH)_8_(NO_3_)_2_·2H_2_O – ZHN (monoclinic structure, space group C2/m, JCPDS 72–0627) with a calculated average crystallite size higher than 100 nm. This sample was labelled ZHNMn. Similar results concerning different reaction products associated with the modification of the synthesis temperature have been reported in ref.^47^.

According to literature^47–51^, there are several possible thermal decomposition paths of bulk ZHN into ZnO, all of them at temperatures below 200 °C. All reports point to a sequential process involving different intermediary compounds and mechanisms, depending on the synthesis conditions and post-synthesis treatments, expected to affect also the dopant distribution. The thermal decomposition of the ZHNMn sample to ZnO was monitored by XRD and is represented in Fig. 1(b), which shows diffraction patterns measured after isochronal annealing at selected temperatures. After isochronal annealing up to 130 °C, the XRD pattern reveals the presence of three crystalline phases: about 50% ZnO, 40% Zn_3_(OH)_4_(NO_3_)_2_ and 10% Zn_5_(OH)_8_(NO_3_)_2_·2H_2_O. After further isochronal annealing up to 170 °C, two crystalline phases in almost equal quantities are observed, namely Zn_3_(OH)_4_(NO_3_)_2_ and ZnO. Finally, after isochronal annealing up to 200 °C the decomposition of the ZHNMn sample into nano-ZnO (average crystallite size 50 ± 3 nm) is complete. Only a barely detectable amount (about 1%) of ZnOHNO_3_·H_2_O with poor crystallinity was identified (Fig. S1 in Supplementary Information). A similar pattern was reported for the thermal decomposition of Zn_5_(OH)_8_(NO_3_)_2_·2H_2_O in ref.^49^.

### Transmission electron microscopy (TEM) results

**Figure 2:**
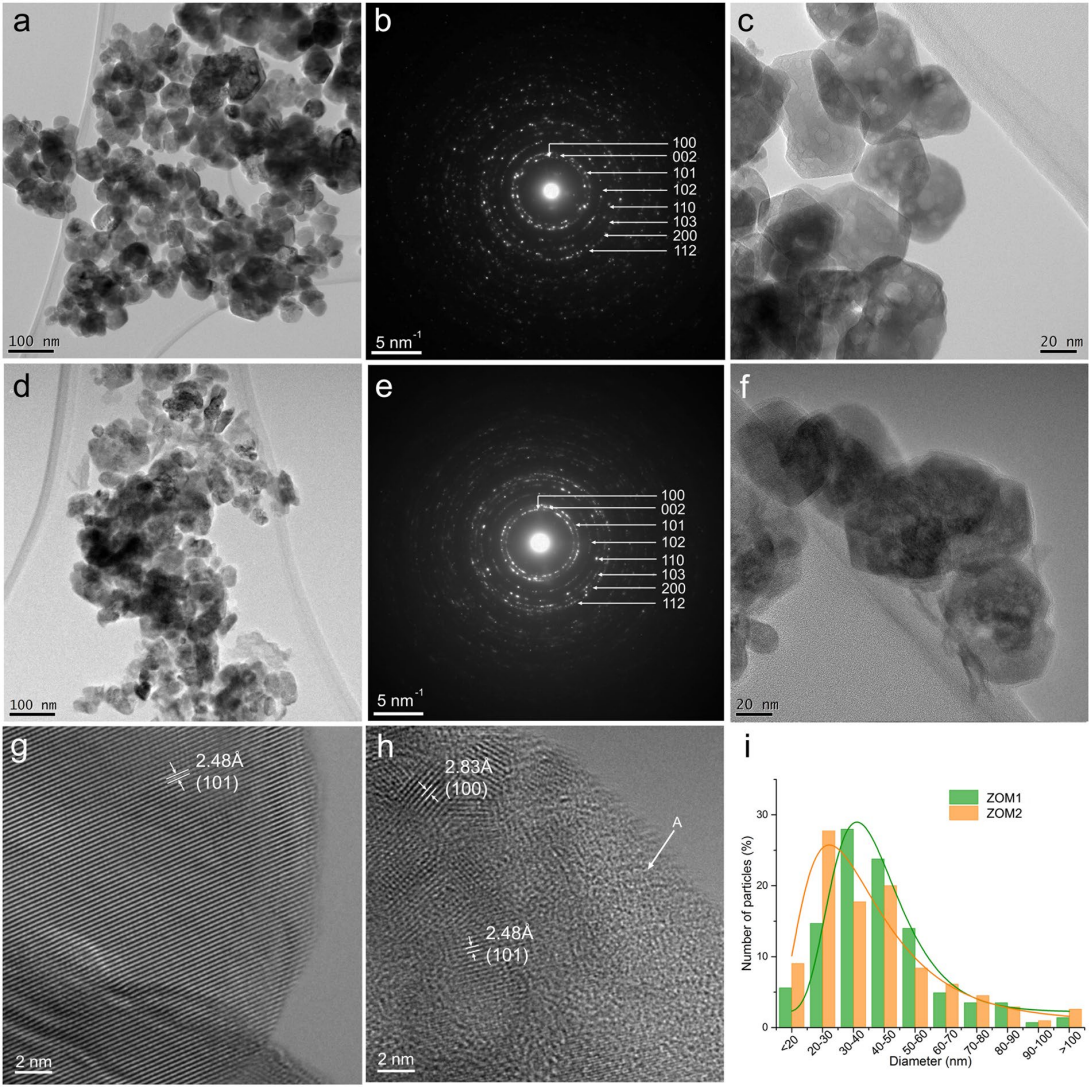
TEM images at low magnification (**a**,**d**), at high magnification (**c**,**f**), SAED patterns (**b**,**e**), HRTEM images (**g**,**h**), and nanocrystals size distributions (**i**) fitted with the log-normal function (thin lines) of the ZOM1 and ZOM2 samples, respectively.

TEM images of the ZOM1 (Fig. 2a) and ZOM2 (Fig. 2d) samples show the formation of polyhedral nanocrystals. Facets are more clearly observed for the nanocrystals of the ZOM1 sample, as seen in both low and high magnification images in Fig. 2. The Selected Area Electron Diffraction (SAED) patterns (Fig. 2b,e) demonstrate that in both samples the nanocrystals structure is hexagonal, with the space group P6_3_mc. The particle size distribution for the ZOM1 sample (Fig. 2i) has a log-normal shape, with a most likely value (mode) around 41 nm and a standard deviation of 20 nm. A similar distribution has been determined for the ZOM2 sample with the most likely value 32 nm and a standard deviation of 21 nm. The obtained results are in line with the XRD results. The slight deviation from the size values provided by XRD is due to the chosen sampling rate (10 nm) in building the histogram and the lower statistical relevance when counting the nanocrystals in the TEM micrographs.

In the high magnification image from Fig. 2f we notice the presence of a thin amorphous layer partially coating the nanocrystals of the ZOM2 sample. The presence of this amorphous shell explains the lower definition of the observed nanocrystal facets and the lower contrast of the images in Fig. 2d,f. The high magnification images from Fig. 2c,f reveal also the porosity of the nanocrystals in these two samples.

The high resolution TEM (HRTEM) images of ZOM1 (Fig. 2g) and ZOM2 (Fig. 2h) confirm the difference of crystallization degree between the two samples. Lattice fringes can be noticed running to the very edge of the grains for the ZOM1 sample, while the ZOM2 sample shows a lower degree of crystallinity and the presence of amorphous material (marked with A in Fig. 2h).

**Figure 3:**
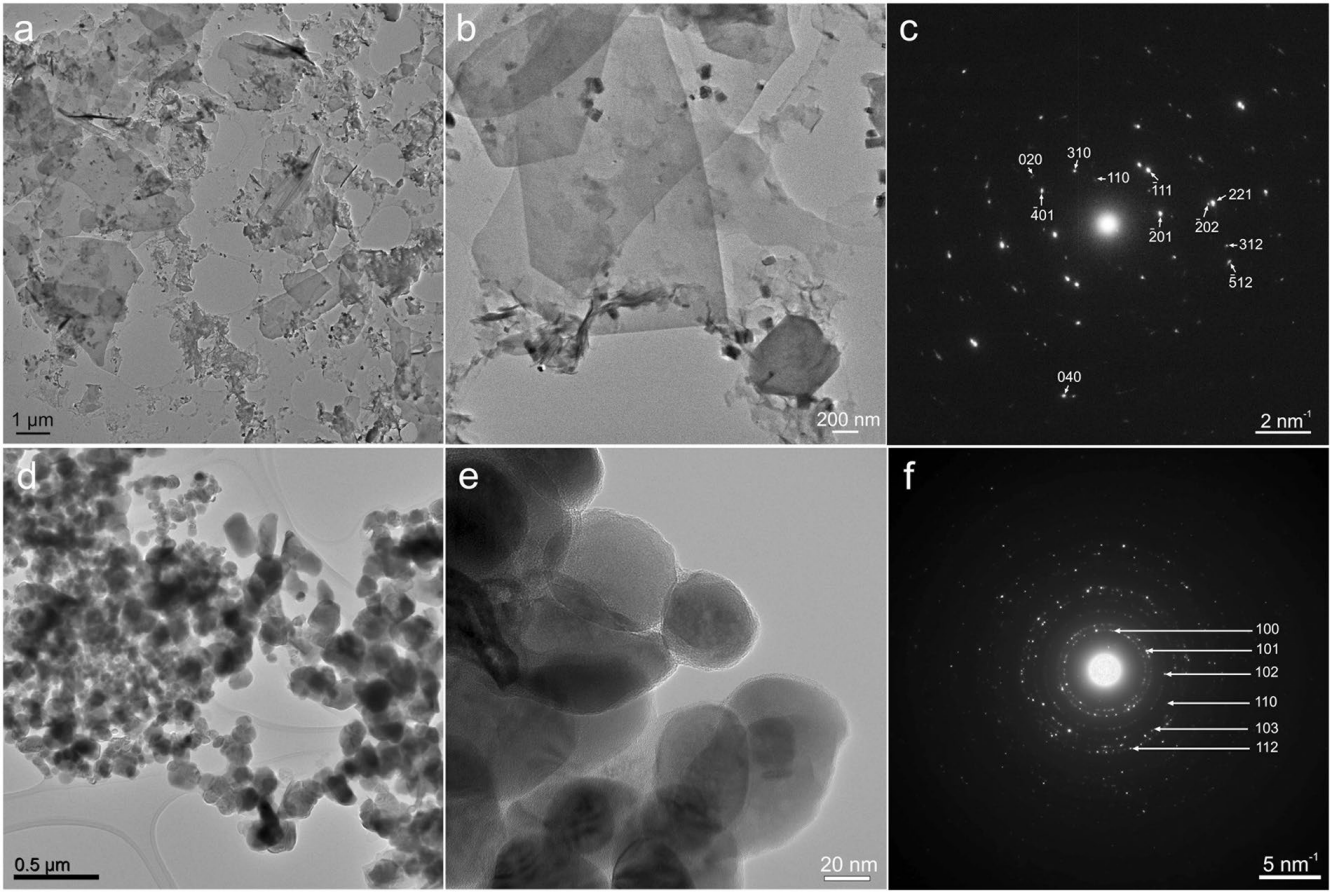
TEM images at low and high magnifications of the ZHNMn sample, as-prepared (**a**,**b**) and after isochronal annealing at 200 °C (**d**,**e**). The corresponding SAED patterns were indexed with the Zn_5_(OH)_8_(NO_3_)_2_·2H_2_O structure for the as-prepared sample (**c**) and with the ZnO wurtzite structure after isochronal annealing at 200 °C (**f**).

The TEM investigation of the ZHNMn sample (Fig. 3a,b) has revealed a particular morphology consisting of large sheet-like (lamellar) structures, with areas in the 0.2 to 6 µm^2^ range. The SAED pattern (Fig. 3c) recorded on such a sheet-like structure corresponds to Zn_5_(OH)_8_(NO_3_)_2_·2H_2_O crystallized in monoclinic structure with C2/m space group. It is important to emphasize here that observing the ZHNMn sample in the as-prepared status was possible only by working in low-beam conditions. The increase of the electron irradiation dose, like for working in HRTEM conditions, lead to the full transformation of ZHNMn into ZnO:Mn.

After isochronal annealing at 200 °C, the ZHNMn sample transformed into ZnO wurtzite as demonstrated by the SAED pattern in Fig. 3f. The ZnO grain size ranges from a few tens of nm up to 200–250 nm (Fig. 3d,e). The thin amorphous layer observed at high magnifications on the surface of the ZnO nanocrystals (Fig. 3e) could be related to the ZnOHNO_3_·H_2_O minority phase observed in the XRD pattern of this sample (Fig. S1 in Supplementary Information), considering the rapid amorphization of the zinc hydroxynitrate induced under an intense electron beam at high magnifications.

### FTIR results

**Figure 4:**
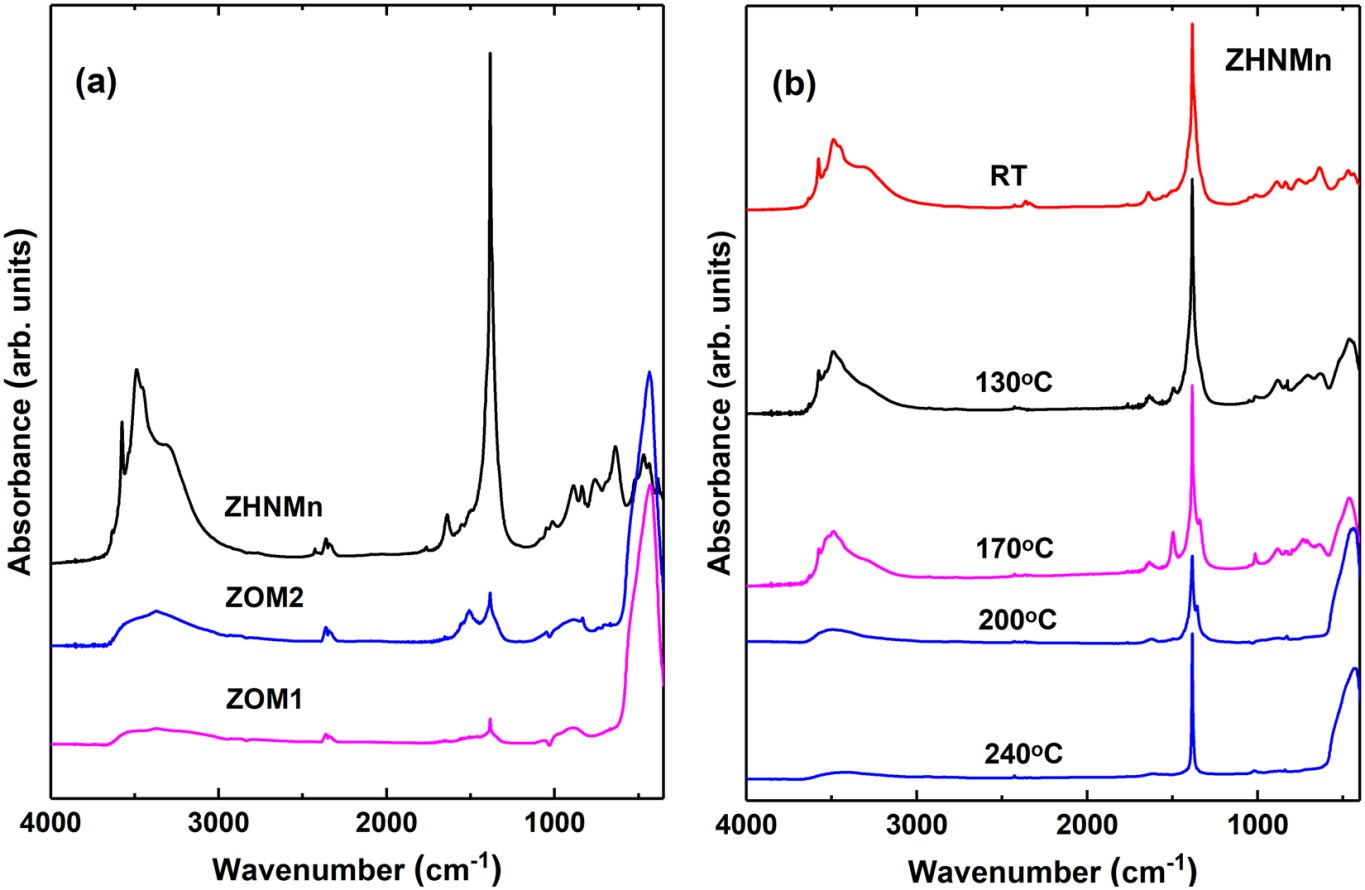
(**a**) FTIR spectra of the investigated samples. (**b**) FTIR spectra of the ZHNMn sample, as-prepared (RT) and isochronally annealed at the indicated temperatures.

The FTIR spectra of the three as-prepared samples show absorption bands with similar positions and shapes, but quite different relative intensities, depending on the relative concentrations of the various phases in each sample (Fig. 4a).

For the ZOM1 and ZOM2 samples, the FTIR spectra present a strong band at ~460 cm^−1^ characteristic to the stretching vibration mode of the Zn-O bond in ZnO, indicating that the dominant phase in these samples is ZnO. The band position and the lack of splitting assert the absence of shape anisotropy in the ZnO nanocrystals morphology^52^. The very weak absorption bands observed in the fingerprint region can be ascribed to the nitrate NO_3_^−^ ion, namely a peak at 1385 cm^−1^ and a broad band in the spectral range 1000–800 cm^−1^ for both samples, as well as an additional band at 1500 cm^−1^ for the ZOM2 sample^53,54^. This points to the presence of a minority phase containing nitrate in the ZOM1 and ZOM2 samples. The broad asymmetric absorption band observed in the spectral range 3600–3200 cm^−1^ is assigned to the hydroxyl groups from minority phases and the -OH groups of adsorbed water molecules^53–56^.

The FTIR spectrum of the ZHNMn sample is similar with the reported infrared spectra of the Zn_5_(OH)_8_(NO_3_)_2_·2H_2_O compound^47,50,54,55,57,58^. The sharp peak around 3570 cm^−1^ and the strong peak at 3400 cm^−1^ were assigned to various stretching vibrations of the O-H bonds in Zn_5_(OH)_8_(NO_3_)_2_·2H_2_O. The broad band around 3300 cm^−1^ and the peak around 1640 cm^−1^ indicate the presence of water molecules in the interlayer space of this layered hydroxide salt or adsorbed on the surface. In the spectral range 1500-800 cm^−1^ there are several well defined absorption bands characteristic to the nitrate group. Thus, the most intense peak around 1380 cm^−1^ corresponds to the ν_3_ vibration (NO_2_ stretching mode of a free nitrate ion), while the weak peaks around 1050 cm^−1^, 840 cm^−1^ and 720 cm^−1^ correspond to the ν_1_ (NO stretch of a free nitrate group), ν_2_ (out-of-plane vibration) and ν_4_ (NO_2_ bend) vibrations, respectively. The presence of only a very weak and broad band at 1050 cm^−1^, as well the fact that the absorption band at around 1380 cm^−1^ is not split, indicate that the nitrate ions are not coordinated to the central Zn ions in the ZHNMn sample. This is indeed expected for the layered structure of Zn_5_(OH)_8_(NO_3_)_2_·2H_2_O^50,55^. The weak shoulder at ~1500 cm^−1^ was associated to the partial dehydration of Zn_5_(OH)_8_(NO_3_)_2_·2H_2_O^55^. This assignment applies to the 1500 cm^−1^ band observed in the ZOM2 spectrum too.

Figure 4(b) displays the FTIR spectra of the isochronally annealed ZHNMn sample at selected temperatures. Important changes in the peaks shape, position and intensity, as well as the appearance of new peaks can be observed in the spectra corresponding to the various temperatures. For the sample annealed at 130 °C the broad band at 3300 cm^−1^ is almost absent, showing that the crystallization water molecules were removed, while the weak band at 1640 cm^−1^ proves that the surface water is still present. The weak peak at 1500 cm^−1^ was assigned to the asymmetric stretching vibration mode for the nitrate ion in zinc hydroxynitrate with the structural formula Zn_3_(OH)_4_(NO_3_)_2_, where the nitrate ion is coordinated directly to the metallic centre^55^. The small shoulder observed around 1330 cm^−1^ is also assigned to the coordinated nitrate, in agreement with the structure of Zn_3_(OH)_4_(NO_3_)_2_. The medium absorption band observed around 460 cm^−1^ is assigned to the Zn-O bond in ZnO^52^. These results, in good agreement with the XRD data, indicate that in the sample annealed at 130 °C three phases co-exist: Zn_5_(OH)_8_(NO_3_)_2_·2H_2_O, Zn_3_(OH)_4_(NO_3_)_2_ and ZnO. The annealing of the ZHNMn sample at 170 °C resulted in an increase in the intensity of the absorption bands of the nitrate ion around 1500 cm^−1^ and 1050 cm^−1^. These changes are in agreement with the formation of the Zn_3_(OH)_4_(NO_3_)_2_ as a major phase, along with the ZnO phase, as observed from the XRD analysis. After annealing at 200 °C, FTIR indicates the presence of ZnO as a predominant phase, as well as the presence of yet another secondary phase containing hydroxide and nitrate groups, which according to the XRD data corresponds to the minority phase ZnOHNO_3_·H_2_O. This is supported by the splitting of the 1380 cm^−1^ band (ν_3_ vibration mode of the nitrate ion) observed in the FTIR spectra of the sample annealed at 170 and 200 °C^55^. The FTIR spectrum of the sample annealed at 240 °C contains an absorption maximum around 450 cm^−1^, characteristic to the ZnO predominant phase with an isotropic morphology of the nanocrystals. Also, around 1380 cm^−1^ an intense narrow peak is observed, characteristic to the free nitrate ion. However, given the shape and intensity of this band, it is more likely that it belongs to NO_2_ gas molecules captured on the surface of the ZnO nanocrystals, as a result of the decomposition of the zinc hydroxynitrates^50^. This narrow peak is also observed with lower intensity, superimposed on a broader peak in all as-prepared samples (Fig. 4a), due to the nature of the precursors and synthesis conditions.

### EPR results

**Figure 5:**
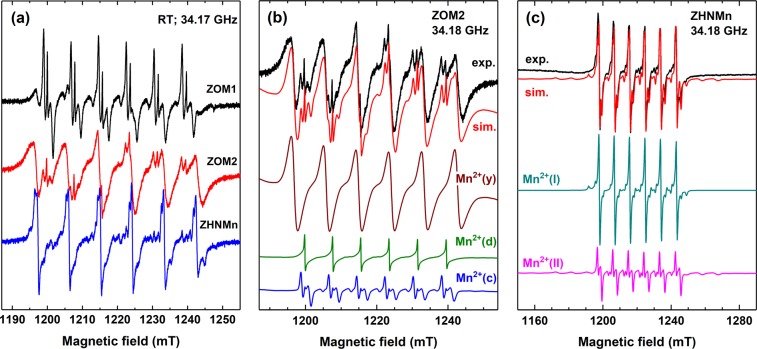
(**a**) EPR spectra of the as-prepared samples measured at RT. (**b**,**c**) Experimental (exp.) and simulated (sim.) EPR spectra of the ZOM2 and ZHNMn samples, respectively. The component spectra of the indicated centres, calculated with the SH parameters from Table S2, are presented below with their relative intensities.

The EPR spectra of the as-prepared samples are presented in Fig. 5(a). For each sample the EPR spectrum consists of several overlapping individual spectra with a characteristic six line hyperfine structure, attributed to Mn^2+^ ions (*S* = 5/2, *I* = 5/2) localized in different host lattices (see Supplementary Information).

In the case of the ZOM1 sample, the EPR spectrum is dominated by six groups of close lying lines with a complex pattern, with a ~7.8 mT separation between the groups (Supplementary Information Fig. S2), typical for Mn^2+^ ions substitutionally localized in Zn^2+^ sites in ZnO^59^. This complex sextet is generated by two manganese centres, called Mn^2+^(c) and Mn^2+^(d), consisting of Mn^2+^ ions localized in ZnO nanocrystals and disordered ZnO, respectively (Supplementary Information Fig. S2). The additional sextet of weaker lines with a larger separation (~9.0 mT) belongs to a third centre called Mn^2+^(x), corresponding to Mn^2+^ ions localized in a host lattice different from ZnO that we denoted “phase x”. The spin Hamiltonian (SH) parameters of the three Mn^2+^ centres are listed in Supplementary Information Table S2.

The analysis of the Mn^2+^ paramagnetic centres in the ZOM1 sample shows that 63% of the Mn^2+^ ions are localized in the ZnO host lattice (Table 1), the remaining 37% being hosted in the minority phase x. The EPR spectrum of ZOM1 is very similar in all details with the spectrum reported for a nano-ZnO:Mn sample obtained by a similar procedure, but without adding nitric acid^15^. For that previously reported sample the x phase was assumed, based on FTIR results, to consist of rests of nitrate compounds from precursors^15^.

**Table 1:** Distribution of the Mn^2+^ Paramagnetic Centres in the As-Prepared and Thermally Annealed ZOM1 and ZOM2 Samples^a^.

Sample	ZnO		Minority phase	
	Mn^2+^(c)	Mn^2+^(d)	Mn^2+^(x)	Mn^2+^(y)
ZOM1	44%	19%	37%	—
ZOM1–200 °C	81%	19%	—	—
ZOM2	17%	4%	—	79%
ZOM2–250 °C	58%	23%	—	19%

^a^Estimated accuracy of Mn distribution is ± 4%.

The EPR spectrum of the ZOM2 sample resembles the spectrum of the ZOM1 sample, although the intensities of the EPR lines attributed to the Mn^2+^ ions in ZnO are very weak. The spectrum is dominated by a sextet of very intense EPR lines, with a line separation of ~9.2 mT, from yet another paramagnetic centre that we denoted Mn^2+^(y) (Fig. 5b). The best simulation of the experimental spectrum (Fig. 5b) has been obtained considering the presence of the paramagnetic centres Mn^2+^(c) and Mn^2+^(d) in ZnO and Mn^2+^(y) in a disordered “phase y”, with SH parameters listed in Table S2 in Supplementary Information.

The analysis of the Mn^2+^ paramagnetic centres in the ZOM2 sample shows that only ~21% of the Mn^2+^ ions are localized in ZnO (Table 1). As ~79% of the Mn^2+^ ions in the ZOM2 sample are localized in the disordered y phase, it is important to determine its composition by further analysing the EPR properties of the Mn^2+^(y) centre. As observed in the TEM image of the as-prepared ZOM2 sample (Fig. 2f), the ZnO nanocrystals are surrounded by an amorphous shell which is most probably a mixture of the disordered y and ZnO phases. Given the very small amount of y phase, the local concentration of the Mn^2+^(y) centres is expected to be high enough to determine increased magnetic dipolar interactions between the Mn^2+^ ions, which would explain the broad EPR lines of the Mn^2+^(y) spectrum.

As the synthesis procedure is similar for the ZOM2 and ZHNMn samples, except for the synthesis temperature, we would assume that the y phase is related to ZHN. Moreover, as shown in Fig. 4(a), the FTIR spectrum of the ZOM2 sample contains features which could be associated with the presence of a small amount of ZHN. In order to confirm this hypothesis, the EPR parameters of the Mn^2+^(y) centres should be similar with the EPR parameters of the Mn^2+^ ions in ZHNMn. From structural investigations two cationic sites were identified in Zn_5_(OH)_8_(NO_3_)_2_·2H_2_O, one with the Zn^2+^ ions octahedrally coordinated by six OH^−^ groups and one with Zn^2+^ ions tetrahedrally coordinated by three OH^−^ groups and one water molecule^51,60^. Figure 5(c) displays the deconvolution of the EPR spectrum of the ZHNMn sample, where two paramagnetic centres, Mn^2+^(I) and Mn^2+^(II), were identified. The SH parameters determined for the Mn^2+^(I) centres (Table S2 in Supplementary Information) have a very low anisotropy and are quite close to the EPR parameters of Mn^2+^ in brucite^61^, which points to a localization of the Mn^2+^ ions in the octahedrally coordinated cationic sites in ZHN. The SH parameters of the Mn^2+^(II) centres indicate a local crystal field with axial symmetry, which could correspond to Mn^2+^ ions localized in the tetrahedrally coordinated cationic sites. To the best of our knowledge, this is the first report of the EPR parameters of Mn^2+^ in ZHN. These parameters can be further used as reference data, especially as there is a growing scientific interest in the investigation of brucite-like layered hydroxide salts (including ZHN) due to both ion-exchange property and intercalation capacity^47,48,53,57,62,63^.

As seen in Table S2 in Supplementary Information, the EPR parameters determined for the Mn^2+^(y) centres in the ZOM2 sample are close to the parameters of the two Mn^2+^ centres in ZHNMn. The Mn^2+^(y) spectrum could be well reproduced by a summation of the spectra calculated with the Mn^2+^(I) and Mn^2+^(II) parameters, considering a disordered host lattice (see Supplementary Information) and broad lines (Δ*B* = 1.0 mT). This result supports the assignation of the Mn^2+^(y) spectrum to Mn^2+^ ions localized in a disordered Zn_5_(OH)_8_(NO_3_)_2_·2H_2_O shell surrounding the ZnO nanocrystals.

### EPR of the thermally annealed samples

**Figure 6:**
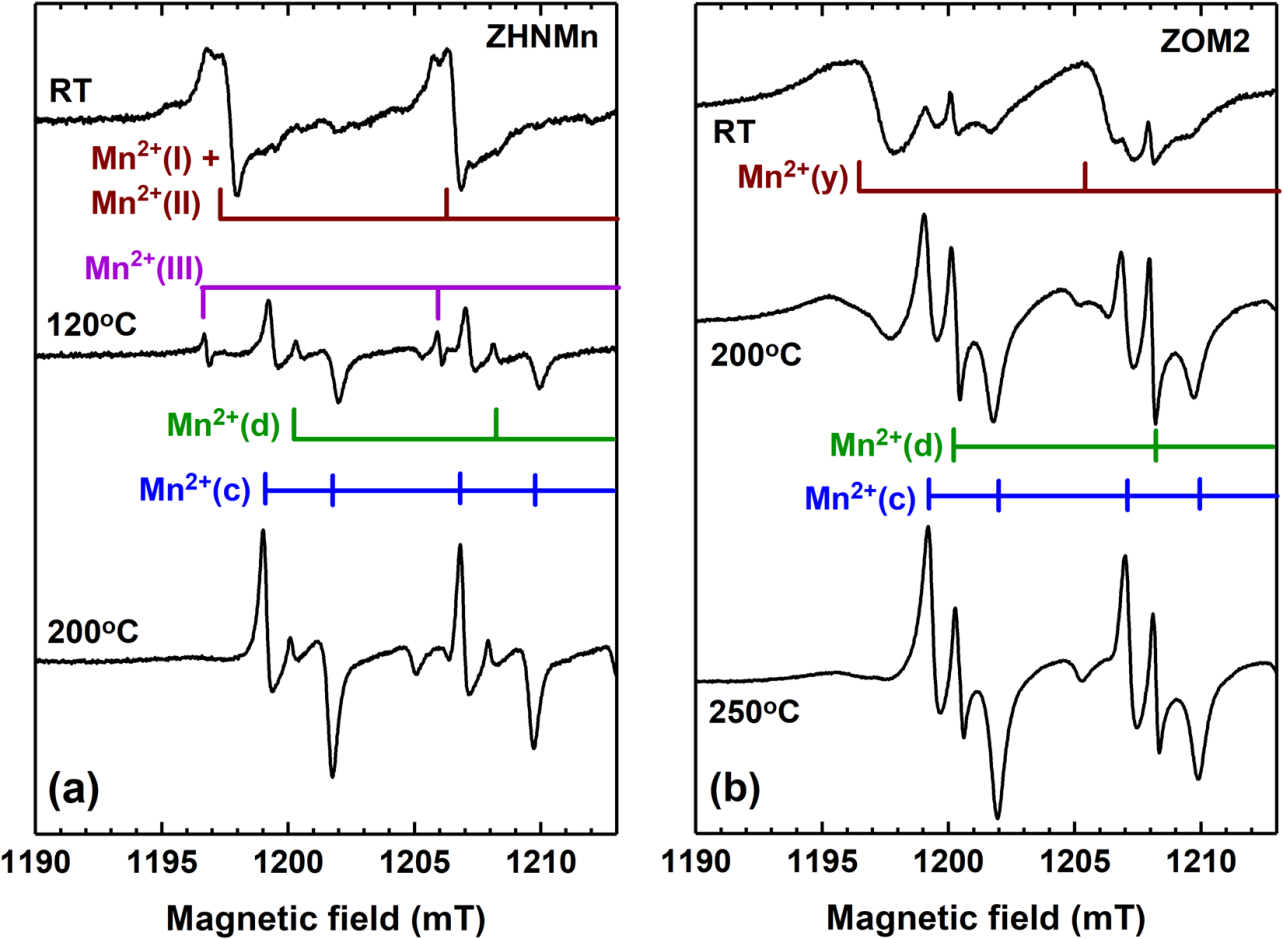
Detailed view of selected EPR spectra of the Mn^2+^ ions in the as-prepared ZHNMn and ZOM2 samples (RT) and isochronally annealed at the indicated temperatures. For all spectra only the first two hyperfine transitions at low-field are displayed.

Further information was obtained by comparing the behaviour under annealing of the ZHNMn and ZOM2 samples, as reflected in the evolution of the corresponding EPR spectra versus the isochronal annealing temperature. As presented in Fig. S3a,b in Supplementary Information, the dominant EPR spectrum of the Mn^2+^ ions in the as-prepared samples gradually transforms into the characteristic spectrum of Mn^2+^ ions in ZnO, following the annealing induced transformation of the host lattice into ZnO. We have selected from Fig. S3a,b three spectra measured before annealing, after a significant intermediate annealing step and at the end of the annealing process. A detailed view of these spectra is displayed in Fig. 6, in order to facilitate the identification of the Mn^2+^ centres and follow their transformation under annealing in the two samples.

By annealing the ZHNMn sample at temperatures above 100 °C the Mn^2+^(I) and Mn^2+^(II) EPR spectra become weaker, disappearing completely at 120 °C (Figs S3a and 6a). Also, new centres are formed above 100 °C. The evolution of the Mn^2+^ centres reflects the evolution of their host material, namely the decomposition of ZHNMn and the formation of new phases. Two of the new Mn^2+^ centres are the Mn^2+^(c) and Mn^2+^(d) centres in ZnO (Figs 6a and S4 in Supplementary Information). The other newly formed Mn^2+^ centres, called Mn^2+^(III), exhibit very narrow lines with a larger line separation (~9.4 mT), and are visible in the EPR spectra of the annealed sample up to 170 °C. These centres could correspond to Mn^2+^ ions localized substitutionally in Zn^2+^ cationic sites in the intermediate Zn_3_(OH)_4_(NO_3_)_2_ phase, identified by XRD. In this compound the cationic sites are sixfold coordinated by four OH^−^ and two NO_3_^−^ groups, which are expected to create a local crystal field with higher anisotropy than in the case of the Mn^2+^(I) centres coordinated by six OH^−^ ^63^.

After the 170 °C annealing step all Mn^2+^ ions are localized in ZnO (Fig. S3a in Supplementary Information), even if, according to XRD, the amount of ZnO in the sample represents only about 50% (Fig. 1b). This result suggests that the transformation of the Zn_5_(OH)_8_(NO_3_)_2_·2H_2_O into ZnO is initiated at lower temperatures in the sample regions containing manganese than in the pure ones. The EPR spectrum of the ZHNMn sample annealed at 200 °C contains only the lines of the Mn^2+^(c) centres, showing that all Mn^2+^ ions are now localized in the core of the ZnO nanocrystals.

The EPR broadening parameter of the Mn^2+^(c) centre in ZnO is correlated with the crystallite size, following an empirical law^64^. According to this relationship, the crystallite size of the ZnO nanocrystals formed by the thermal decomposition of the ZHNMn sample at 200 °C would be around 52 nm (see Supplementary Information for more details). This value perfectly matches the crystallite mean size resulted from the XRD analysis of this sample. This is a very important result, proving the *uniform distribution* of the Mn^2+^ ions *inside* the ZnO nanocrystals resulted from the thermal decomposition of ZHNMn.

In the case of the ZOM2 sample, the thermally induced transformation of the Mn^2+^(y) centres into both Mn^2+^(c) and Mn^2+^(d) centres proceeds at a slower pace, their spectrum being still visible at 250 °C (Figs S3b and 6b). No other types of Mn^2+^ centres are formed during annealing. We can infer therefore that the complete decomposition into ZnO of the disordered Zn_5_(OH)_8_(NO_3_)_2_·2H_2_O phase hosting the Mn^2+^(y) centres takes place at higher temperatures than for the ZHNMn sample, without the formation of intermediate compounds. We assume that this behaviour is due to the size (a few atomic layers) and disordered state of the ZHNMn shell surrounding the ZnO nanocrystals. Such a size induced disparity in the thermal behaviour was previously observed in the case of the disordered nanometric ε-Zn(OH)_2_ shell on ZnS nanocrystals, which decomposed into ZnO at higher temperatures and in different steps than polycrystalline ε-Zn(OH)_2_^19^.

The broadening parameter of the Mn^2+^(c) centres spectrum in ZOM2 which, according to ref.^64^, would correspond to a crystallite size of ~25 nm, does not vary across the whole annealing range. In fact, the Mn^2+^(c) spectrum does not change with annealing, except for increasing in intensity. After the final annealing step at 250 °C the distribution of the Mn^2+^ ions in ZOM2 changed to 81% localized inside the ZnO lattice (centres Mn^2+^ (c) and Mn^2+^ (d)), and 19% embedded in the remaining ZHNMn (Table 1). We can infer thus that most of the initial ZHN amorphous shell thermally transformed into ZnO.

The EPR spectra of the ZOM1 sample submitted to isochronal thermal annealing up to 200 °C (Supplementary Information Fig. S3c) show the disappearance of the Mn^2+^(x) lines at 170 °C due to the thermal decomposition of the x phase into ZnO. Indeed, after the final annealing step at 200 °C the Mn^2+^ ions are all localized inside ZnO (Table 1). The broadening parameter of the Mn^2+^(c) spectrum did not change after annealing. Given that the amount of Mn^2+^(d) centres remained practically the same after annealing and that the crystallization process of ZnO necessitates higher temperatures/annealing times^65^, we assume that the Mn-rich ZnO resulting from the decomposition of the x phase was added directly as crystallized layers on the existing ZnO nanocrystals.

## Discussion

The characteristics of the investigated samples, before and after annealing, demonstrate that minor alterations of the preparation conditions could lead to nanostructured materials with significantly modified properties, fit for a wide range of different applications.

The as prepared ZOM1 sample has 44% of the dopant ions uniformly distributed in the ZnO nanocrystals core, 19% are localized in the disordered ZnO phase, while 37% are found in rests of precursors. The thermal annealing up to 200 °C affects only to a very small extent the sample structure, as the secondary phase detected in the as-prepared sample is in a very small amount. The thermally induced decomposition of this phase into ZnO leads to a quite interesting distribution of the Mn^2+^ ions in the ZnO nanocrystals after the final annealing step, with 37% of them embedded in the outer layers of the nanocrystals, besides the 44% uniformly distributed through the core. Given that the activation energy for the Mn^2+^ diffusion in bulk ZnO is 276.9 kJ/mol^66^, an intra-crystalline diffusion process of the Mn^2+^ ions from the outer layers into the core of the ZnO nanocrystals is not likely to take place at the low annealing temperatures used in our experiments. Indeed, previous investigations on a nanostructured ZnO film showed that, after annealing at 600 °C for one hour, the Mn^2+^ ions segregated at the grain boundaries in the as-prepared film partially diffused into the peripheral regions of the ZnO nanocolumns^16^. This result proves that even at a higher temperature the main active process is the diffusion in interfaces/grain boundaries. The non-uniform dopant distribution in the annealed ZOM1 sample could therefore be expected to be quite stable. The porous structure of the nanocrystals increases the effective surface of the sample, making it interesting for surface based applications (e.g. gas sensors).

On the other hand, for the as-prepared ZOM2 sample, consisting of smaller size ZnO porous nanocrystals, most of the dopant ions (79%) are localized in a minority phase of amorphous ZHN surrounding the ZnO nanocrystals and only 17% of them are found in the ZnO core, according to EPR. After the last annealing step at 250 °C, 41% of the Mn^2+^ ions are added to the outer layers of the ZnO nanocrystals, while some of the nanocrystals are still covered by a mixture of disordered ZnO and ZHN. These results are supported by the structural information from the TEM-HRTEM images of the ZOM2 sample after annealing at 250 °C. Figure S5 in Supplementary Information shows a mixture of well crystallized nanocrystals (Fig. S5b) and nanocrystals covered with an amorphous shell (Fig. S5c). The shell is most probably a mixture of disordered ZnO and the remaining ZHNMn, as identified in the corresponding EPR spectrum. The XRD analysis of this sample shows no significant increase of the crystallite size after annealing. Given that in the as-prepared sample the amorphous ZHN phase formed a rather thin layer on the ZnO nanocrystals, a significant increase in the ZnO nanocrystals size by annealing could not be expected. The results obtained in the case of the ZOM2 sample show that, by applying a mild thermal treatment, one can modulate the dopant distribution by making use of the intermediary ZHN phase. The ability to functionalize ZnO nanocrystals with a doped shell with tailored amount and distribution of the dopant is interesting for surface based applications as gas sensors and catalysis.

The 200 °C annealed ZHNMn sample consists of ZnO nanocrystals with better crystallinity (Fig. 3e,f), with all dopant Mn^2+^ ions *uniformly distributed* inside the ZnO lattice. The relatively low temperature (200 °C) at which the uniformly doped ZnO nanocrystals are formed by this synthesis route is interesting for applications, given that the growth of ZnO nanocrystals of such size from a pure disordered ZnO phase would need temperatures above 500 °C^64^. Such nanocrystals could be interesting for applications relying on volume properties, like optoelectronics. ZnO nanocrystals with uniform Mn^2+^ distribution were previously obtained from the thermal decomposition of hydrozincite, but at higher temperatures^67^. Our results show that the thermal decomposition of doped precursors at *low temperatures* (below 300 °C) is a reliable, simple and cost-effective method for the preparation of uniformly doped nano-ZnO:Mn.

In summary, we have investigated the effect of slight changes in the synthesis procedure on the dopant distribution in nanocrystalline ZnO doped with Mn^2+^ ions in low concentration prepared by co-precipitation. By varying one synthesis parameter from one sample to another we obtained two nano-ZnO:Mn samples with different dopant distributions and minority phases and a polycrystalline Mn-doped zinc hydroxynitrate sample (ZHNMn). The minority phases from the two nano-ZnO:Mn samples were identified as residues of precursor nitrate compounds in the ZOM1 sample and ZHN in the ZOM2 sample.

In the as-prepared ZHNMn sample the Mn^2+^ ions were substitutionally localized in the two types of Zn^2+^ sites. The isochronal annealing treatment up to 200 °C lead to the complete transformation of the ZHNMn sample into nanocrystalline ZnO, with all Mn^2+^ ions uniformly distributed in the core of the ZnO nanocrystals.

A very different situation was encountered for the as-prepared ZOM2 sample: only 21% of the Mn^2+^ ions were incorporated in ZnO, while 79% of them were localized in the minority ZHN phase, present as a thin amorphous layer around the ZnO nanocrystals. This is already an interesting result, showing the possibility to functionalize the ZnO nanocrystals with ZHN, a layered material with various applications^47^. The thermal annealing process up to 250 °C lead to the decomposition of three quarters of ZHN directly into ZnO, with part of it adding as crystalline layers on the initial nanocrystals and the rest remaining as disordered ZnO. After the last annealing step the ZnO nanocrystals contained 58% of the Mn^2+^ ions, 17% uniformly distributed in the core and 41% in the outer layers.

The distribution pattern of the Mn^2+^ ions in the annealed ZOM1sample is an intermediate case, with a smaller difference between the core and surface concentrations than in the ZOM2 sample.

Our results demonstrate a strategy to tailor the dopant distribution in colloidal ZnO nanocrystals by small adjustments of the preparation algorithm by a simple and cost-effective method. Thus, the most appropriate route to obtain a uniform dopant distribution in the ZnO nanocrystals volume seems to be the thermal decomposition of a doped precursor, while a non-uniform distribution with dopant-rich layers at the nanocrystals surface can be obtained from the thermal decomposition of minority phases. ZnO nanocrystals covered by a dopant-rich shell are of interest for surface sensitive applications like catalysis or gas-sensing, while uniformly doped ZnO:Mn nanocrystals are suitable for applications in nanoelectronics, optoelectronics or spintronics. These findings are expected to contribute to a better understanding of the doping mechanisms at nanoscale and to increase the applicative opportunities.

## Methods

### Synthesis

Several samples of nano-ZnO doped with 0.1 at% nominal concentration of Mn^2+^ ions were prepared by co-precipitation using the same precursors and steps for all samples. An aqueous solution containing zinc nitrate (0.19 M) and manganese nitrate (0.02 M), corresponding to 0.1 at% Mn^2+^ nominal concentration, has been prepared at 80 °C and subsequently acidified with HNO_3_ 1 M and magnetically stirred for several minutes. The amounts of HNO_3_ 1 M added to the different samples were in the range of 50 to 250 mL in steps of 50 mL. Afterwards, this mixture was precipitated with a strong basic solution of NaOH 2.4 M (in excess) added slowly with a peristaltic pump, under continuous magnetic stirring. The pH values at the end of the syntheses varied from 10 to 5 for the corresponding 50 to 250 mL HNO3 1 M additions. The suspension thus obtained was left to age for one hour at the same temperature, under continuous magnetic stirring. The precipitate was collected by centrifugation and washed several times with bi-distilled water and absolute ethanol, then air dried at 60 °C.

Another sample was prepared following the same procedure with the maximum amount of HNO_3_ 1 M of 250 mL, but lowering the synthesis temperature to 35 °C. The XRD investigation identified this sample as zinc hydroxynitrate [Zn_5_(OH)_8_(NO_3_)_2_·2H_2_O].

### Investigation techniques

XRD investigations were performed with a Bruker D8 Advance X-ray diffractometer provided with a Cu anode and Ni filter (*λ* = 1.54184 Å), in the *θ* − *θ* geometry. The lattice parameters of the ZnO crystalline phases and the average crystallites size were determined with the Topas v.3 software (Bruker) by Rietveld refinement.

TEM studies were carried out using the JEOL JEM ARM200F and JEOL 2100 electron microscopes operated at 200 kV. The samples were observed in conventional and high resolution TEM (TEM/HRTEM) modes in order to gain information regarding the shape and size of the nanograins as well as the likely presence of secondary phases. The ZHNMn sample was investigated in low-beam conditions to avoid the amorphization or transformation into ZnO.

FTIR spectra were recorded with a Spectrum BX II (Perkin Elmer) spectrometer in the 4000-350 cm^−1^ spectral range, by accumulating 128 scans at a resolution of 4 cm^−1^. The powdered samples were diluted into KBr powder in a 1:100 mass ratio, ground thoroughly and pressed into pellets.

EPR investigations were performed on powder samples inserted in calibrated pure fused-silica tubes of 2 mm inner diameter. Q*-*band (34 GHz) EPR measurements were performed at room temperature (RT) with the Bruker E500Q spectrometer from the Centre for advanced ESR/EPR techniques (CetRESav - http://cetresav.infim.ro/). The spin Hamiltonian (SH) parameters of the paramagnetic centres were determined with the EASYSPIN v.5.1.11 software^68^.

Isochronal annealing experiments from 100 °C up to 250 °C were performed in air in 10 °C steps in order to study the thermal evolution of the samples. The sample was annealed at each set temperature in a temperature stabilized (±1 °C) furnace for 10 min. and then cooled down to RT for complementary measurements by XRD, EPR and FTIR.

## Supplementary information

Supplementary Information

## Data Availability

All data generated or analysed during this study are included in this manuscript (and its Supplementary Information file).
