# Supplementary material for: Tailoring the Dopant Distribution in ZnO:Mn Nanocrystals
Source: Sci Rep. 2019 May 3;9:6894. doi: 10.1038/s41598-019-43388-z (PMC6499810; doi:10.1038/s41598-019-43388-z)
Supplement: Supplementary file 1 — Supplementary Information [file 41598_2019_43388_MOESM1_ESM.pdf]

Supplementary Information

Tailoring the Dopant Distribution in ZnO:Mn Nanocrystals

Daniela Ghica[+], Ioana D. Vlaicu[+], Mariana Stefan*, Valentin A. Maraloiu, Alexandra C. Joita, Corneliu Ghica

National Institute of Materials Physics, Atomistilor Str. 405A, Magurele, 077125, Romania

* corresponding author

[+] equal contributions

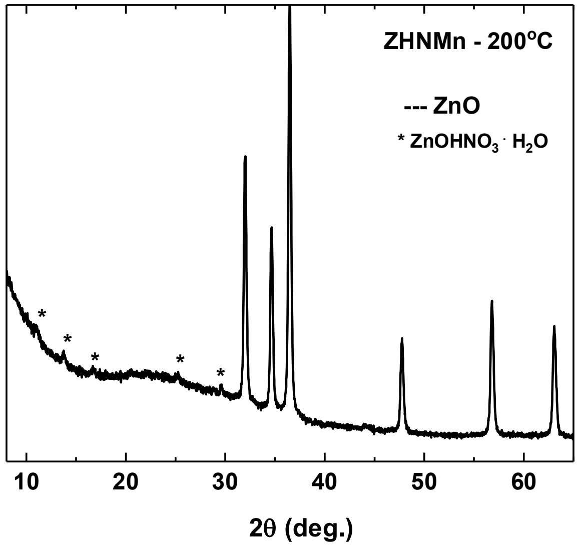

Figure S1. XRD pattern of the ZHNMn sample annealed at 200 °C, showing the transformation into ZnO as main crystallographic phase as well as very low intensity peaks from the $ZnOHNO_3 \cdot H_2O$ minority phase (stars). The very broad XRD line in the 20 – 30 degrees range corresponds to the glass support.

Table S1. Samples selected for in-depth spectroscopic and microstructural investigations: synthesis temperature and amount of HNO3 1M added to the starting solution; composition and crystallite size resulted from XRD at the end of the co-precipitation synthesis; maximum annealing temperature T_{max}; composition and crystallite size after annealing at T_{max}.

Sample	Synthesis temperature	Amount of HNO3 1M	XRD - before annealing	T_{max}	XRD - post annealing at T_{max}
**ZOM1**	80 °C	50 mL	ZnO<br>d = 38 ± 3 nm	200 °C	ZnO<br>d = 38 ± 3 nm
**ZOM2**	80 °C	250 mL	ZnO<br>d = 25 ± 3 nm	250 °C	ZnO<br>d = 26 ± 3 nm
**ZHNMn**	35 °C	250 mL	ZHN<br>d > 100 nm	200 °C	ZnO<br>d = 50 ± 3 nm<br>+ $ZnOHNO_3 \cdot H_2O$ (<1%)

Spin Hamiltonian and analysis of the EPR spectra of the Mn^{2+} centres

The EPR spectra of all three samples under investigation contained overlapping individual spectra from at least two paramagnetic centres associated with isolated Mn^{2+} ions localized in different host lattices. The individual spectra of the paramagnetic centres had different line separations and characteristics such as line intensity, width and shape. For each centre the EPR parameters were determined by lineshape analysis and fitting of the experimental spectrum, using the EasySpin program. The low doping level (0.1 at%) in the three samples under study ensured a decreased magnetic dipolar broadening of the lines and a quite good resolution of the EPR spectra.

The amplitudes of the calculated spectra of the different centres were adjusted for an optimum fit of the experimental spectrum. The simulated spectra of each sample were obtained by summation of the calculated spectra of all centres present in the sample. The intensity of the EPR spectrum of a centre, calculated by double integration of the spectrum, is proportional to the amount of that centre in the measured sample. The relative concentration of a Mn^{2+} centre in a sample was determined as the ratio between the intensity of the corresponding calculated spectrum and the intensity of the simulated spectrum of the sample.

The EPR spectra of the Mn^{2+} paramagnetic centres were analysed using the following spin Hamiltonian (SH):[1]

$$H = \mu_B \mathbf{S} \cdot \mathbf{g} \cdot \mathbf{B} + \mathbf{S} \cdot \mathbf{A} \cdot \mathbf{I} + \frac{a}{6}\left[\left(S_x^4 + S_y^4 + S_z^4\right) - \frac{1}{5}S(S+1)\left(3S^2 + 3S - 1\right)\right] + D\left[S_z^2 - \frac{1}{3}S(S+1)\right] - \mu_N g_N \mathbf{B} \cdot \mathbf{I}$$

The first term represents the main electron Zeeman interaction of the $S = 5/2$ electron spin with the external magnetic field B. The second term represents the hyperfine interaction of the electron spin with the $I = 5/2$ nuclear spin of the ^{55}Mn (100% abundance) isotope, responsible for the characteristic six-lines hyperfine structure. The next two zero-field-splitting (*ZFS*) terms describe the interaction of the electron spin with the local crystal field, which gives rise to a fine structure of $2S = 5$ component lines, while the last term describes the nuclear Zeeman interaction.

For nanocrystalline powders the characteristic features of the EPR spectra are wiped out by spatial averaging and the strong line broadening due to crystal lattice disorder. The most affected are the anisotropic non-central transitions M_S: ±1/2 ↔ ±3/2 and M_S: ±3/2 ↔ ±5/2. The contribution of random strains and lattice disorder can be very important in nanostructured materials, resulting in a variation in the local crystal field at the paramagnetic ion sites, both in magnitude and orientation, reflected in the broadening of the individual EPR line width. The line broadening is reproduced in the lineshape simulation of the experimental spectra by including a statistical distribution of the axial *ZFS* parameter value *D*, with the broadening parameter described by the corresponding standard deviation σ(*D*). The broadening parameter σ(*D*) is expressed as percentage of the *D* parameter value. For a disordered phase σ(*D*) = 43%*D*.

The SH parameters of the Mn^{2+} centres, determined by simulation and lineshape fitting of the Q-band spectra of the investigated samples, are given in Table S2, together with reference SH parameters for the Mn^{2+} ions in other host lattices of interest. For the *ZFS* parameters the absolute values are given, although a prediction of the parameter signs is possible based on the reference data. For example, in the case of the Mn^{2+} ions in ZnO single crystals, the sign of the *D* parameter value was determined from low temperature measurements to be negative[2]. It is thus likely that the sign of *D* is negative in the case of the Mn^{2+} ions in nanocrystalline and disordered ZnO as well.

<u>As-prepared ZOM1 sample</u>

Figure S2 displays the deconvolution of the EPR spectrum of the as-prepared ZOM1 sample. The two centres associated with the Mn^{2+} ions embedded in ZnO nanocrystals, i.e. Mn^{2+}(c) and in the disordered phase, Mn^{2+}(d), have very close EPR parameters, the main difference being the value of the broadening parameter. As initial parameters in the simulation of both centres we have used the reported EPR parameters of the Mn^{2+} ions in the ZnO single crystal, and then adjusted them until the best fit of the experimental spectra was achieved.

The effect of the broadening parameter on the aspect of the EPR spectrum is quite dramatic. The spectrum of the Mn^{2+}(c) centres, where the broadening effect is moderate, can be described as consisting of six lines with a complex shape, each one extending over a magnetic field range ΔB, measured as the distance between the maximum and the minimum of the

complex line (see Figure S2), which is further called "powder linewidth". The powder line shape and ΔB strongly depend on the *D* parameter value, allowing thus the estimation of this parameter from the lineshape fitting of the experimental spectrum. The disorder induced broadening effect leads to the decrease of ΔB, until only six symmetric lines associated with the central allowed M_S:-1/2 ↔ +1/2, Δm = 0 transitions can be seen, as is the case for the spectrum of the Mn^{2+}(d) centres. In this case the *D* value was assumed to be the same as for the Mn^{2+}(c) centres, while the *g* and *A* parameter values could be determined with a good accuracy. The very small differences (less than 2%) in the hyperfine parameters of these two centres can be explained by slightly different average coordination numbers of the Mn^{2+} ions in the disordered phase associated with small variations in sample stoichiometry[3-5].

A similar disorder effect can be observed in the spectrum of the Mn^{2+}(x) centres. In this case a range of possible values was determined for the *D*-parameter for which the spectrum lineshape and width were reasonably well reproduced.

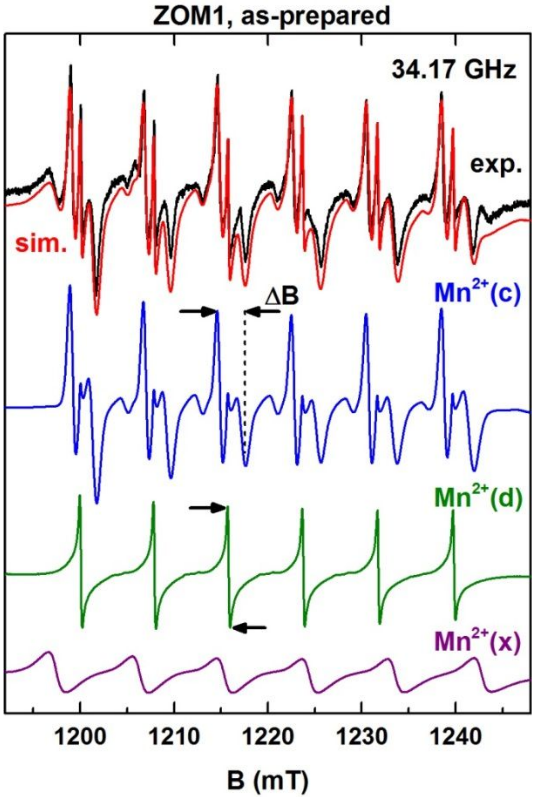

Figure S2. Experimental (exp.) and simulated (sim.) EPR spectrum of the as-prepared ZOM1 sample. The component spectra of the three Mn^{2+} centres calculated with the SH parameters from Table S2 are represented below. The arrows mark the powder linewidth ΔB for the Mn^{2+}(c) and Mn^{2+}(d) centres.

Table S2. The EPR parameters (*g, A, D* and *a*), broadening parameter σ(*D)* and individual peak-to-peak linewidth (ΔB_{pp}) of the Mn^{2+} centres in the investigated samples. Reference parameters of the Mn^{2+} centres in other host materials discussed in the text are given for comparison.

Host	Centres	*g*	*A* [10^{-4} cm^{-1}]	|*D*| [10^{-4} cm^{-1}]	|*a*| [10^{-4} cm^{-1}]	σ(*D*) [%*D*]	ΔB_{pp} [mT]
ZOM1 as-prepared	Mn^{2+}(c)<br>Mn^{2+}(d)<br>Mn^{2+}(x)	2.0012 ± 0.0001<br>2.0011 ± 0.0001<br>2.0012 ± 0.0002	-74.0 ± 0.1<br>-74.3 ± 0.1<br>-84.7 ± 0.1	238 ± 2<br>238 ± 2<br>150-240	-<br>-<br>-	10 ± 1<br>43<br>43	0.12<br>0.12<br>1.0
ZOM2 as-prepared	Mn^{2+}(c)<br>Mn^{2+}(d)<br>Mn^{2+}(y)	2.0012 ± 0.0001<br>2.0011 ± 0.0001<br>2.0011 ± 0.0002	-74.0 ± 0.2<br>-74.3 ± 0.2<br>-85.8 ± 0.5	238 ± 3<br>238 ± 3<br>-	-<br>-<br>-	13 ± 1<br>43	0.2<br>0.15<br>1.0
ZHNMn as-prepared	Mn^{2+}(I)<br>Mn^{2+}(II)	2.0011 ± 0.0002<br>2.0009 ± 0.0002	-84.7 ± 0.2<br>-85.5 ± 0.5	18 ± 2<br>225 ± 5	9 ± 2<br>-	-<br>6 ± 1	0.65<br>0.5
ZHNMn annealed 110°C	Mn^{2+}(c)<br>Mn^{2+}(d)<br>Mn^{2+}(III)	2.0012 ± 0.0001<br>2.0011 ± 0.0001<br>2.0011 ± 0.0001	-74.0 ± 0.1<br>-74.3 ± 0.1<br>-87.5 ± 0.2	238 ± 2<br>238 ± 2<br>-	-<br>-<br>-	7 ± 1<br>43	0.13<br>0.15<br>0.15
ZnO[a]	Mn^{2+}(c)<br>Mn^{2+}(d)<br>Mn^{2+}(x)	2.0012 ± 0.0001<br>2.0011 ± 0.0001<br>2.0012	-74 ± 0.1<br>-74.3 ± 0.1<br>-84.7	238 ± 2<br>238 ± 2<br>150-240	-<br>-<br>-	11<br>43<br>43	0.12<br>0.12<br>1.0
$Zn_{1-x}Mn_xO$ single crystal x = 0.035[b]	Mn^{2+}	2.0012	-73.4	225	|*a-F*|= 5.5	3	-
$Mg(OH)_2$[c]	Mn^{2+}	$g_{\parallel}$ = 2.0001<br>$g_{\perp}$ = 2.0005	$A_{\parallel}$ = -85.7<br>$A_{\perp}$ = -84.9	7.2	10.82	-	-

[a] Ref. 4

[b] Ref. 2

[c] Ref. 6

A previous in-depth study on Mn^{2+} doped ZnO nanocrystals prepared by different methods has shown that the broadening parameter, and, by extension, the degree of lattice disorder, is correlated with the crystallite average size *d* determined by XRD, according to an empirical relationship $\sigma(D) = 4.44 + 34.62 \exp(-d/d_0)$, where $d_0 = 18.34$ nm.[3] According to this relationship, the $\sigma(D) = 9.5$ value used in the simulated spectrum of the Mn^{2+}(c) centres in ZOM1 would correspond to $d \sim 36$ nm, while $\sigma(D) = 13$ determined for Mn^{2+}(c) centres in ZOM2 would give $d \sim 25$ nm. In the case of the Mn^{2+}(c) centres in the ZHNMn sample annealed at 110 ^{o}C, the $\sigma(D) = 6.5$ value used in the simulation (Figure S4) would correspond to a crystallite size of about 52 nm.

As-prepared ZHNMn sample

In the case of the ZHNMn sample, the larger size of the crystallites lead to a lower degree of disorder and, therefore, of strain induced line broadening. The fine structure was discernible in the EPR spectra of the Mn^{2+}(I) and Mn^{2+}(II) centres, allowing the determination of the *ZFS* parameter values (Figure 5c from the manuscript).

The *ZFS* parameters for the Mn^{2+}(I) centres were determined using the EPR parameters reported for the Mn^{2+} ions in brucite[6] as initial values and adjusting them to fit the experimental fine structure. The *D*-parameter value of Mn^{2+} in brucite was negative, while the *a* parameter was positive. The EPR spectrum of the Mn^{2+}(I) centres could be simulated well only with *ZFS* parameters of opposite signs, either the same as in brucite or with the reverse signs.

In the case of the Mn^{2+}(II) centres, low intensity non-central transitions could be observed at low and high magnetic field, and also a complex pattern in the central part of the spectrum, quite similar with the pattern observed for the Mn^{2+}(c) centres. The fitting of these features lead to the estimation of the *D* parameter value.

Thermally annealed samples

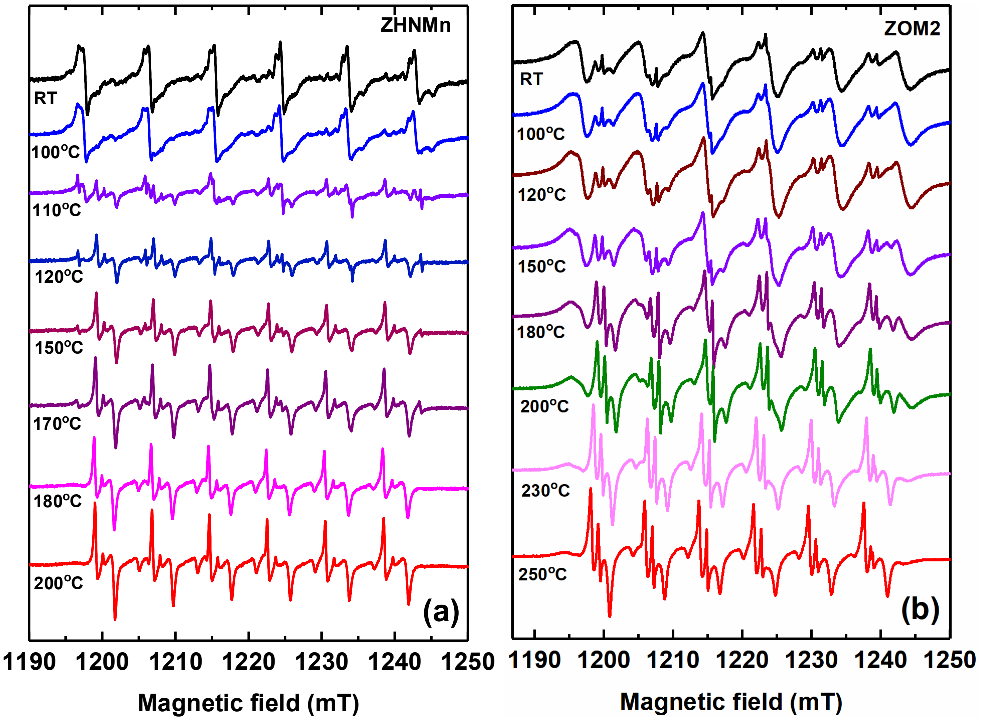

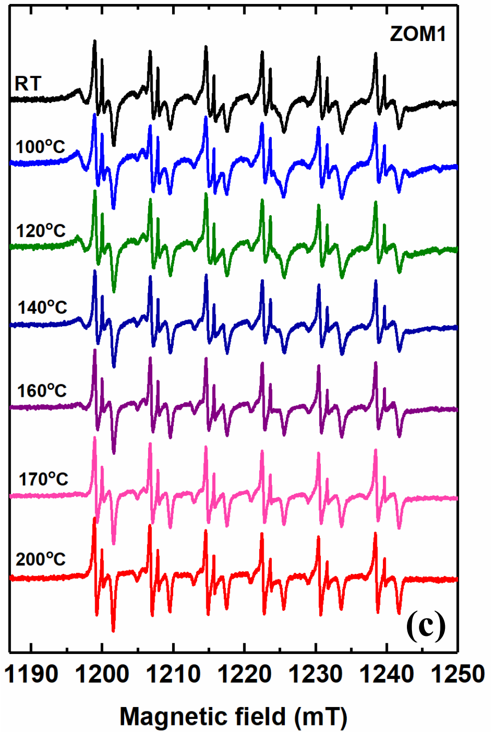

Figure S3. Q-band EPR spectra of the Mn^{2+} ions in the three investigated samples isochronally annealed at the indicated temperatures and measured at RT.

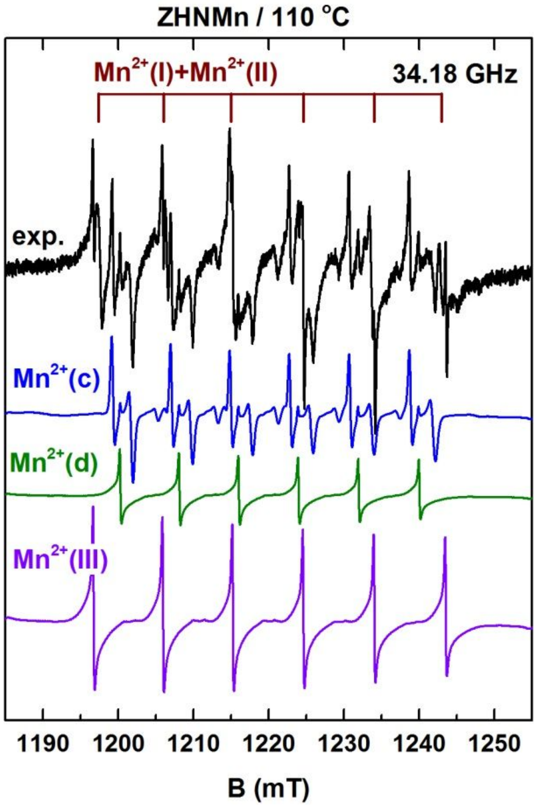

Figure S4. EPR spectrum of the Mn^{2+} ions in the ZHNMn sample isochronally annealed up to 110 °C and measured at RT. The calculated spectra of the three Mn^{2+} centres formed after annealing are represented below. The positions of the EPR lines of the Mn^{2+} ions in $Zn_5(OH)_8(NO_3)_2 \cdot 2H_2O$ (the Mn2+(I) and Mn^{2+}(II) centres) are marked above the experimental spectrum.

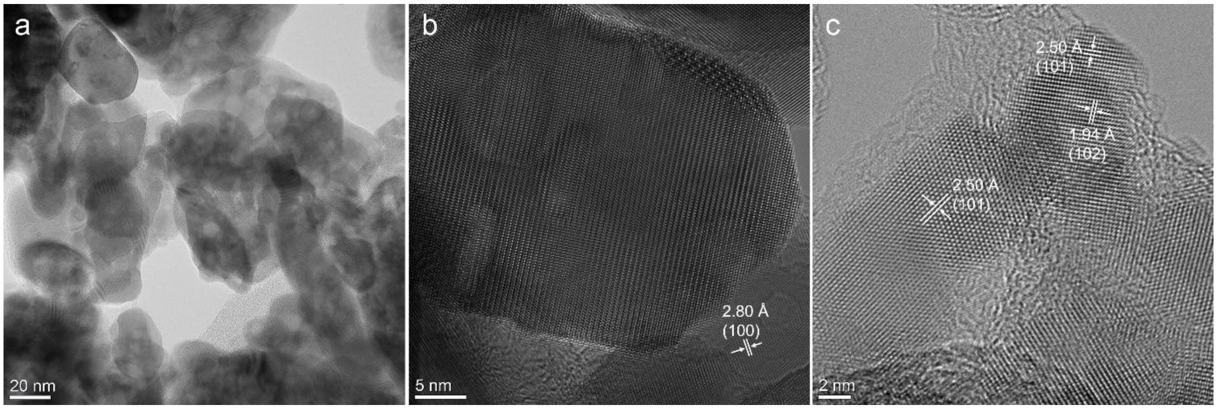

Figure S5. TEM-HRTEM images of the ZOM2 sample after isochronal annealing at 250 °C.

References

(1) Abragam, A. & Bleaney, B. *Electron Paramagnetic Resonance of Transition Ions*; Clarendon Press: Oxford, U.K., 1970.

(2) Diaconu, M. *et al.* Electron paramagnetic resonance of $Zn_{1-x}Mn_xO$ thin films and single crystals, *Phys. Rev. B: Condens. Matter Mater. Phys.* **72**, 085214(6) (2005).

(3) Stefan, M., Nistor, S. V. & Ghica, D. Correlation of lattice disorder with crystallite size and the growth kinetics of Mn^{2+} doped ZnO nanocrystals probed by electron paramagnetic resonance. *Cryst. Growth Des.* **13**, 1350-1359 (2013).

(4) Simanek, E. & Muller, K. A. Covalency and hyperfine structure constant *A* of iron group impurities in crystals, *J. Phys. Chem. Sol.* **31,** 1027-1040 (1970).

(5) Ghica, D., Vlaicu, I. D., Stefan, M., Nistor, L. C. & Nistor, S. V. On the agent role of Mn^{2+} in redirecting the synthesis of $Zn(OH)_2$ towards nano-ZnO with variable morphology. *RSC Adv.* **6**, 106732-106741 (2016).

(6) Pieczonka, W. A., Petch H. E. & McLay, A. B. An Electron Spin Resonance study of manganese impurity in brucite. *Can. J. Phys.* **39**, 145-157 (1961).
